# Characterization of Extracellular Vesicles Produced by Aspergillus fumigatus Protoplasts

**DOI:** 10.1128/mSphere.00476-20

**Published:** 2020-08-12

**Authors:** Juliana Rizzo, Thibault Chaze, Kildare Miranda, Robert W. Roberson, Olivier Gorgette, Leonardo Nimrichter, Mariette Matondo, Jean-Paul Latgé, Anne Beauvais, Marcio L. Rodrigues

**Affiliations:** a Instituto de Microbiologia Paulo de Góes (IMPG), Universidade Federal do Rio de Janeiro, Rio de Janeiro, Brazil; b Department of Mycology, Institut Pasteur, Paris, France; c Plateforme Protéomique, Unité de Spectrométrie de Masse pour la Biologie (MSBio), Centre de Ressources et Recherches Technologiques (C2RT), USR 2000 CNRS, Institut Pasteur, Paris, France; d Instituto de Biofísica Carlos Chagas Filho, Universidade Federal do Rio de Janeiro, Rio de Janeiro, Brazil; e Centro Nacional de Biologia Estrutural e Bioimagem, Universidade Federal do Rio de Janeiro, Rio de Janeiro, Brazil; f School of Life Sciences, Arizona State University, Tempe, Arizona, USA; g Plateforme de Microscopie Ultrastructurale, Imagepole, Institut Pasteur, Paris, France; h School of Medicine, University of Crete, Heraklion, Crete, Greece; i Instituto Carlos Chagas, Fundação Oswaldo Cruz, Curitiba, Brazil; University of Georgia

**Keywords:** *Aspergillus fumigatus*, conidia, protoplasts, extracellular vesicles, *Aspergillus*

## Abstract

Extracellular vesicles (EVs) are membranous compartments produced by yeast and mycelial forms of several fungal species. One of the difficulties in perceiving the role of EVs during the fungal life, and particularly in cell wall biogenesis, is caused by the presence of a thick cell wall. One alternative to have better access to these vesicles is to use protoplasts. This approach has been investigated here with Aspergillus fumigatus, one of the most common opportunistic fungal pathogens worldwide. Analysis of regenerating protoplasts by scanning electron microscopy and fluorescence microscopy indicated the occurrence of outer membrane projections in association with surface components and the release of particles with properties resembling those of fungal EVs. EVs in culture supernatants were characterized by transmission electron microscopy and nanoparticle tracking analysis. Proteomic and glycome analysis of EVs revealed the presence of a complex array of enzymes related to lipid/sugar metabolism, pathogenic processes, and cell wall biosynthesis. Our data indicate that (i) EV production is a common feature of different morphological stages of this major fungal pathogen and (ii) protoplastic EVs are promising tools for undertaking studies of vesicle functions in fungal cells.

**IMPORTANCE** Fungal cells use extracellular vesicles (EVs) to export biologically active molecules to the extracellular space. In this study, we used protoplasts of Aspergillus fumigatus, a major fungal pathogen, as a model to evaluate the role of EV production in cell wall biogenesis. Our results demonstrated that wall-less A. fumigatus exports plasma membrane-derived EVs containing a complex combination of proteins and glycans. Our report is the first to characterize fungal EVs in the absence of a cell wall. Our results suggest that protoplasts represent a promising model for functional studies of fungal vesicles.

## Abstract (precis)

Fungal cells use extracellular vesicles (EVs) to export biologically active molecules to the extracellular space. In this study, we used protoplasts of Aspergillus fumigatus, a major fungal pathogen, as a model to evaluate the role of EV production in cell wall biogenesis. Our results demonstrated that wall-less A. fumigatus exports plasma membrane-derived EVs containing a complex combination of proteins and glycans. Our report is the first to characterize fungal EVs in the absence of a cell wall. Our results suggest that protoplasts represent a promising model for functional studies of fungal vesicles.

## INTRODUCTION

In the *Aspergillus* genus, 90% of all infections resulting in human aspergillosis are caused by Aspergillus fumigatus, which is the most prevalent mold pathogen in immunocompromised patients (1). A. fumigatus has a multifactorial pathogenic arsenal, which allows this organism to successfully establish disease in different hosts (1–4). Aspergillosis begins with inhalation of asexual conidia followed by fungal morphological transition in the absence of a proper immunological response (1).

Fungi, as seen with many other eukaryotic and prokaryotic organisms, produce extracellular vesicles (EVs) (5–8). EVs were first described in the yeast-like pathogen Cryptococcus neoformans (9). Subsequent studies demonstrated EV production in yeast forms of C. gattii, Histoplasma capsulatum, Candida albicans, C. parapsilosis, Sporothrix schenckii, S. brasiliensis, Paracoccidioides brasiliensis, P. lutzii, Malassezia sympodialis, Saccharomyces cerevisiae, Pichia fermentans, and Exophiala dermatitidis (10–17). In filamentous fungi, the presence of EVs was described previously in the phytopathogens Alternaria infectoria (18) and Fusarium oxysporum *f.* sp. *vasinfectum* (19), in the dermatophyte Trichophyton interdigitale (20), and in the emerging human pathogen Rhizopus delemar (21). Recently, it was also reported that mycelial forms of A. fumigatus produce EVs (22).

A major difficulty in directly addressing the physiological roles of EVs is the lack of understanding of their intracellular biogenesis and of the mechanisms underlying cell wall crossing. An original approach for studying the generation of EVs would be to use protoplasts and look at their active release in the absence of a cell wall. In fact, protoplasts might represent a promising model for the study of EVs, as already suggested by Gibson and Peberdy 50 years ago, who chose the name “subprotoplasts” for the vesicle-like particles budding from the plasma membrane of A. nidulans cells (23).

Our primary goal in the present work was to search for EVs produced by protoplasts of A. fumigatus germinating conidia. Our results revealed the presence of typical EVs in protoplast supernatants incubated under different conditions. EV cargo was directly influenced by the experimental conditions under which A. fumigatus was incubated. Our report provides experimental evidence that fungal EVs are produced not only by the mycelial morphological stage of A. fumigatus, but also by protoplasts of germinating conidia, especially during cell wall regeneration. To our knowledge, this is the first demonstration that protoplasts may represent a useful model to analyze the production and role of EVs in the absence of any cell wall in an experimental setting similar to the ones which allowed the study of exosomes produced by mammalian cells (24).

## RESULTS

### Observation of outer particles resembling EVs in protoplasts of A. fumigatus germinating conidia.

**FIG 1:**
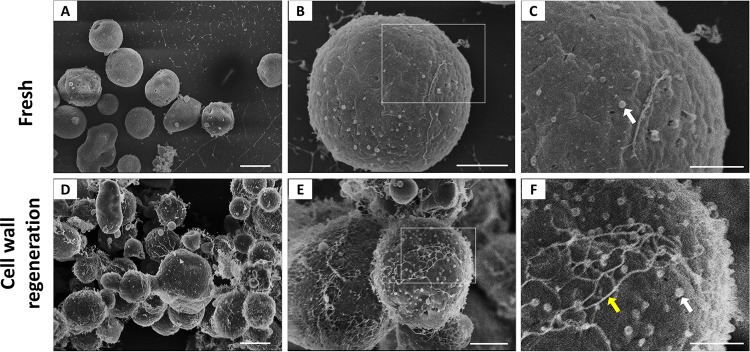
Morphological aspects of freshly prepared and cell wall-regenerating protoplasts. Fresh protoplasts (A to C) and cell wall-regenerating cells (D to F) are shown under conditions of increased magnification by SEM. Panels C and F represent magnified views of the boxed areas in panels B and E, respectively. The magnified views suggested the occurrence of outer particles with properties compatible with EVs (white arrows). Under cell wall-regenerating conditions, a fibril-like network was more abundantly detected (yellow arrow). Scale bars represent 5 μm in panels A and D, 2 μm in panels B and E, and 1 μm in panels C and F. At least 50 cells were analyzed, and the results are representative of at least two independent experiments producing similar morphological profiles. Similar analyses using superresolution SEM produced similar results (data not shown).

To experimentally overcome the difficulties of detecting EVs *in situ* due to the presence of a thick cell wall, we adopted an experimental model using protoplasts. Fungal cells lacking cell walls were obtained by enzymatic digestion with a lysing enzyme from Trichoderma harzianum (Glucanex), which hydrolyzes cell wall components for protoplast preparation (25). These cells can reconstruct their walls in osmotically stabilized media containing 0.6 M KCl, glucose, nitrogen, and salts (26–30). Incubation of protoplasts in 0.6 M KCl only, on the other hand, impairs cell wall synthesis (A. Beauvais and T. Fontaine, unpublished data). In this context, we first compared the morphological aspects of freshly obtained A. fumigatus protoplasts with cell wall-regenerating cells by scanning electron microscopy (SEM). Under both experimental conditions, we observed ∼50-nm-diameter extracellular structures in an apparent association with the fungal surface (Fig. 1). When the protoplasts were incubated under conditions of cell wall synthesis, the outer particles were more numerous, and a fibril-like network was observed.

**FIG 2:**
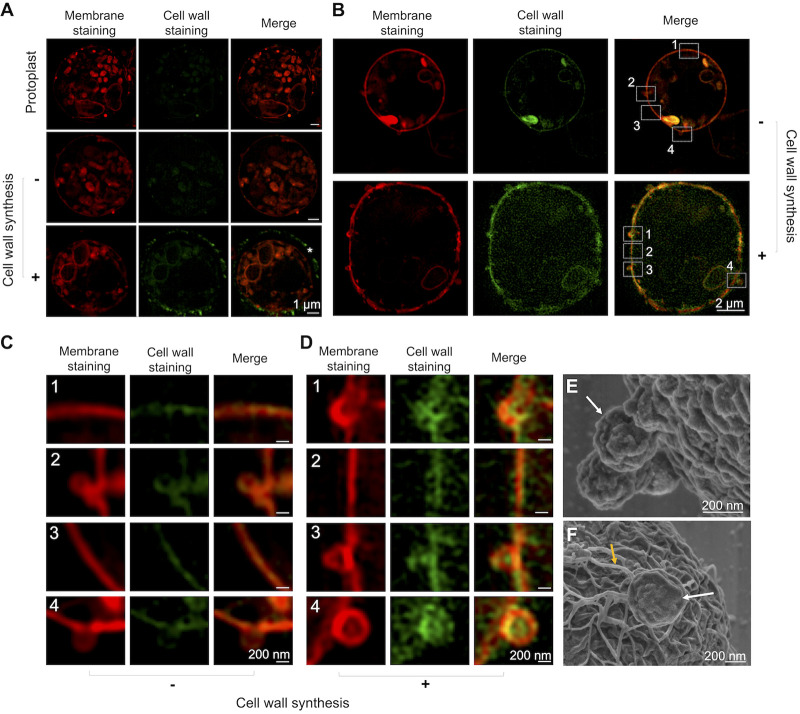
Membrane projections in A. fumigatus protoplasts. (A) Freshly purified protoplasts were stained with DiI, a lipophilic dye (red fluorescence). Cell wall staining with an anti-glucan antibody was at the background levels. Similar results were observed for protoplasts incubated under nonregenerating conditions. During cell wall regeneration (2 h), glucan staining (green fluorescence) was abundant at the cell surface (asterisk). (B) Detailed analysis of nonregenerating and cell wall-regenerating cells revealed an association between glucan staining and outer membrane projections only in cell wall-regenerating protoplasts (90 min of incubation). (C and D) Enhanced views of the boxed areas (numbered 1 to 4) of fungal cells in the absence of cell wall synthesis and under cell wall-regenerating conditions, respectively. (E and F) A detailed view of the surface of protoplasts provided by superresolution SEM confirmed the occurrence of outer particles (white arrows) budding from the plasma membrane in nonregenerating protoplasts (E) and regenerating (2 h) protoplasts (F). Fibrillar material closely associated with the outer membrane projection was uniquely detected during cell wall regeneration (F, yellow arrow). At least 10 cells were analyzed, and the results are representative of two independent experiments producing similar morphological profiles.

To analyze these vesicle-like particles in intact protoplasts, we checked the membrane organization of freshly prepared protoplasts, protoplasts undergoing cell wall synthesis for 2 h (medium with the osmotic stabilizer KCl and nutrients), and protoplasts incubated for 2 h under the starvation condition (in KCl only). Incubation outcomes were monitored by staining the protoplasts with an anti-glucan antibody and observation by fluorescence microscopy. Membranes were stained with the lipophilic dye DiI. A. fumigatus protoplasts manifested the complex membrane distribution that is typically observed in most eukaryotic cells (Fig. 2A). As expected, glucan was detected at the background levels in freshly prepared protoplasts and in protoplasts incubated in KCl alone. Under conditions of cell wall synthesis, surface glucan was unequivocally detected. A detailed analysis of the relationship between membrane staining and glucan detection in these cells revealed several regions of membrane projection to the outer space (Fig. 2B). Under nonregenerating conditions, no glucan was detected (Fig. 2C). During induction of cell wall synthesis, the projected regions were closely associated with glucan detection, and, in fact, the polysaccharide was apparently surrounded by the membranous compartments (Fig. 2D). The occurrence of membrane projections in protoplasts incubated under nonregenerating or cell wall synthesis conditions was confirmed by superresolution SEM (Fig. 2E and 2F, respectively).

### Protoplasts of A. fumigatus germinating conidia release EVs.

**FIG 3:**
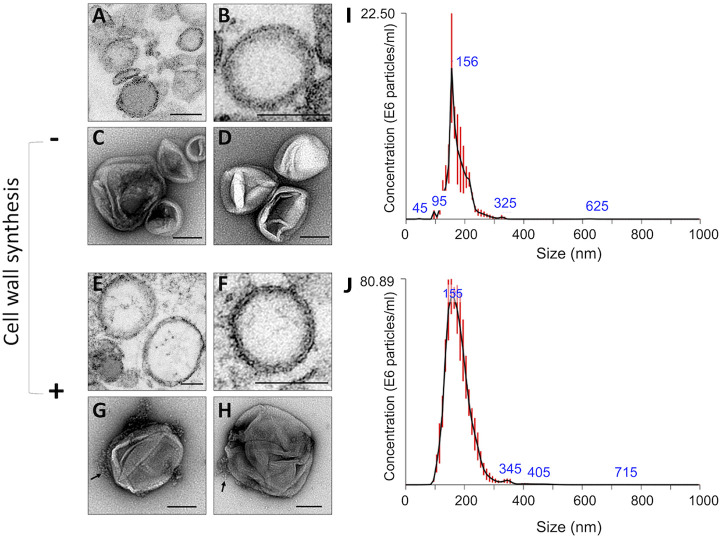
Analysis of EVs obtained from A. fumigatus protoplasts. (A to H) Protoplast EVs were analyzed by regular TEM (A, B, E, and F) or after negative staining (C, D, G, and H). Independent illustrations of each condition are shown for each technique. Under conditions stimulating cell wall synthesis, fibril-like structures associated with EVs were observed (G and H, arrows). The results are representative of at least two independent experiments producing similar morphological profiles. Scale bars correspond to 100 nm. (I and J) NTA of isolated vesicles demonstrated similar distributions of EVs in the 50-to-300-nm-diameter range, independently of the condition of incubation of the protoplasts. NTA was repeated twice, producing similar results.

Fungal preparations used for both qualitative and quantitative EV analyses were adjusted to 10^8^ protoplasts/ml, and cell viability was in the 87% to 93% range throughout all of the experiments. Supernatants obtained from protoplasts incubated under regenerating and nonregenerating conditions were fractionated by ultracentrifugation, and the resulting pellets were analyzed by transmission electron microscopy (TEM). Membranous structures with a typical bilayer and with the morphological aspects and dimensions previously observed for fungal EVs were isolated from protoplast supernatants (Fig. 3A, B, E, and F). Regenerating protoplasts produced EVs that were apparently associated with fibrillar material (Fig. 3G and H; see also Fig. S1 in the supplemental material). Most of the vesicles were in the 200-nm-diameter range, and this visual perception was confirmed by nanoparticle tracking analysis (NTA), which revealed a major peak of vesicle detection in the 150-nm-diameter range (Fig. 3I and J). EVs obtained from control or regenerating protoplasts had very similar diameter distribution profiles.

10.1128/mSphere.00476-20.1 FIG S1 Microscopic analysis of the association between vesicle-like particles and fibrillar material in A. fumigatus protoplasts. (A) Analysis of protoplasts by superresolution scanning electron microscopy of recently prepared cells (fresh) or cells incubated under nonregenerating (non-reg) or regenerating (reg) conditions suggests that surface fibrillar material is associated with particles with dimensions resembling those observed in EVs (arrow). (B) Transmission electron microscopy analysis of EVs isolated under nonregenerating (non-reg) or regenerating (reg) conditions indicates the presence of fibril-like, electron-dense material in association with vesicles. (C) Analysis of EVs obtained under cell wall-regenerating conditions by negative staining confirms the observation of fibril-like material in association with vesicles. Download FIG S1, PDF file, 2.4 MB. Copyright © 2020 Rizzo et al. 2020 Rizzo et al. This content is distributed under the terms of the Creative Commons Attribution 4.0 International license.

### EVs are more abundantly detected in the supernatants of regenerating protoplasts.

**FIG 4:**
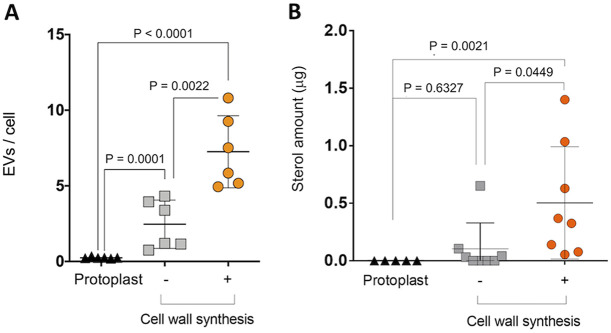
EV quantification during the cell wall synthesis process in A. fumigatus protoplasts. (A) Quantitative NTA of EVs produced by freshly prepared protoplasts and from protoplasts incubated under conditions of cell wall regeneration or nonregeneration. (B) Determination of sterol concentration in EVs obtained from supernatants of fresh protoplasts, nonregenerating protoplasts, and protoplasts incubated under cell wall-regenerating conditions. In panels A and B, values are reported as means ± standard deviations of results obtained from at least two and five independent experiments, respectively. Paired comparisons were statistically analyzed using the *t* test tool in GraphPad Prism 6 software.

We quantified EV production in the experimental systems explored in our study by different approaches. First, independent replicates were submitted to quantitative NTA. This analysis revealed that the EV/cell ratios were at the background levels for the samples from freshly prepared protoplasts (Fig. 4A). Even though some EVs were released over time in the supernatants of control protoplasts, their number was drastically increased in the supernatant of regenerating protoplasts. The NTA data agreed with the quantification of the sterols in the EV-containing supernatants (Fig. 4B). This increase in the sterol levels was not associated with an enhancement in the total amount of the protoplast sterols occurring during cell wall regeneration (data not shown).

### Glycan components of A. fumigatus EVs.

**FIG 5:**
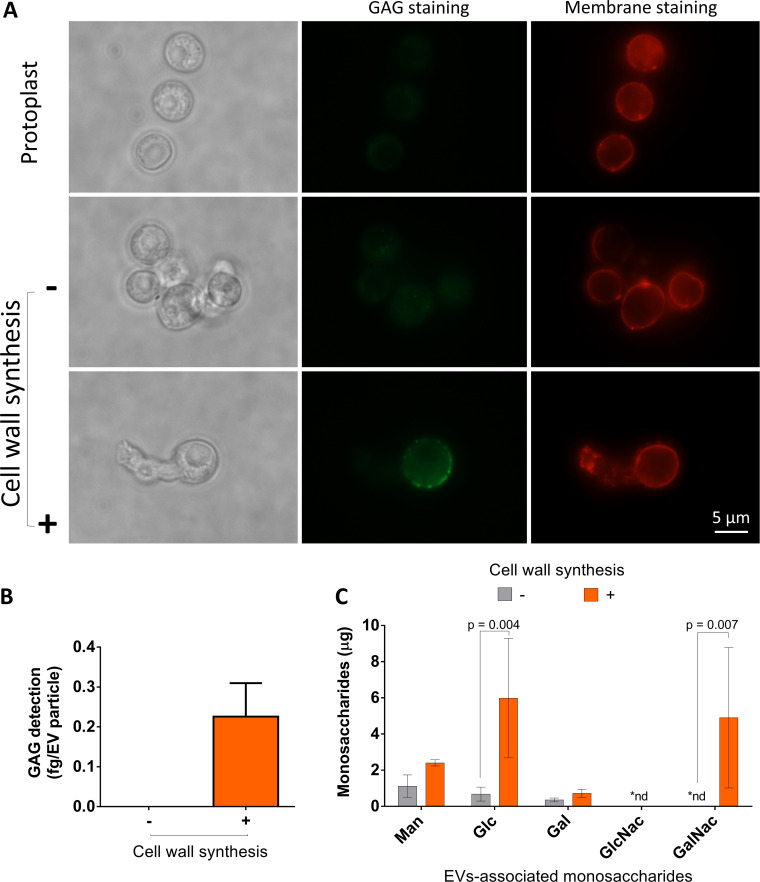
Analysis of glycan synthesis during cell wall regeneration in A. fumigatus conidial protoplasts. (A) Membrane and GAG staining in A. fumigatus protoplasts. All cells were efficiently stained with DiI (red fluorescence). During cell wall synthesis (2 h), GAG was detected in association with the fungal surface. The scale bar corresponds to 5 μm. (B) Serological detection of GAG (ELISA) in EVs obtained from protoplasts. Positive reactions with a GAG-binding antibody were observed only in EVs obtained during cell wall synthesis. (C) Gas chromatography-mass spectrometry (GC-MS) analysis of sugar units of EVs. In agreement with an involvement of EVs in cell wall synthesis, GalNAC (a GAG component) was observed only in EVs obtained from protoplasts during cell wall regeneration. The increased detection of Glc during cell wall synthesis (2 h of germination) is consistent with the presence of EV-associated glucans. The results are representative of two independent replicates producing similar profiles.

The molecular composition of the EVs released by the protoplasts was investigated. Since galactosaminogalactan (GAG) is a marker of polysaccharide secretion by the A. fumigatus mycelium (4), we first showed the presence of this polysaccharide during protoplast regeneration (Fig. 5A). We then proved that GAG was present in EVs isolated from protoplasts incubated under the conditions of cell wall regeneration and was absent in control vesicles (Fig. 5B). These results agreed with the compositional analysis of the carbohydrate units of EVs. Glucosyl (Glc), mannosyl (Man), and galactosyl (Gal) units were detected in EV preparations obtained from both cell wall-regenerating and nonregenerating protoplasts (Fig. 5C). N-Acetyl-galactosaminyl (GalNAc) residues, which are markers of GAG, were found only in regenerating protoplasts. In addition, Glc levels were significantly increased in EV samples from these cells compared to nonregenerating protoplasts (*P* = 0.004). *N*-Acetylglucosamine (GlcNAc) residues were absent in all samples.

### Proteomic analysis of EVs.

Proteomic analysis revealed only 142 proteins in EVs produced by fresh protoplasts, contrasting with the detection of 2,056 proteins in vesicles from regenerating protoplasts (see Table S1 in the supplemental material). All 142 of the EV proteins detected in fresh protoplasts were found in samples obtained under cell wall regeneration conditions. Although EVs were more abundantly detected in the regenerating protoplasts, the qualitative protein composition of the nonregenerating protoplast was similar (Table S1).

10.1128/mSphere.00476-20.2 TABLE S1 Complete list of proteins identified in EVs of A. fumigatus protoplasts. Download Table S1, XLSX file, 0.7 MB. Copyright © 2020 Rizzo et al. 2020 Rizzo et al. This content is distributed under the terms of the Creative Commons Attribution 4.0 International license.

**FIG 6:**
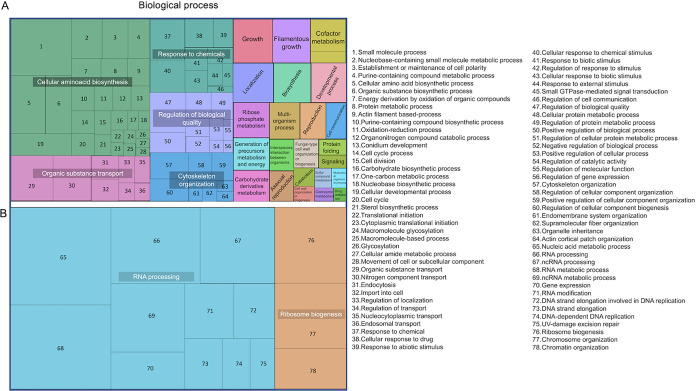
Proteomic analysis of EVs obtained from supernatants of A. fumigatus protoplasts. TreeMap views of all biological processes with which vesicular proteins were associated are presented. Panel A shows the biological processes common to regenerating and nonregenerating conditions. Panel B shows the processes that were exclusively found under conditions of cell wall regeneration. Rectangular areas reflect the *P* value of enrichment of GO terms in the *Aspergillus* database. GO terms are gathered under summarized terms using the REVIGO tool (77). ncRNA, noncoding RNA. Major cellular processes are specified in panels A and B. Subclasses of each cellular process are listed on the figure’s right side.

The predicted GO classification of all EV-related proteins identified numerous terms (Fig. 6). As previously described for several fungal EVs (11, 14, 22, 31), the shared GO terms corresponded to proteins involved in a wide range of processes of fungal physiology. Most of the biological processes (680 GO terms) were common to both the regenerating and nonregenerating conditions. A minor fraction of biological processes (50 GO terms) were specifically found in the cell wall-regenerating system. We used the UniProt (https://www.uniprot.org/) and AspGd (http://www.aspgd.org/) databases to specifically analyze proteins related to cell wall assembly under each of the sets of experimental conditions used in this study (Table 1). We detected several proteins related to (i) cell wall synthases, including the β1,3 glucan synthase Fks1 and its GTPase activator Rho1 (32), α1,3 glucan synthases Ags (33), chitin synthases (34), Ktr mannosyltransferases involved in the synthesis of galactomannan (35), and enzymes belonging to the GAG biosynthetic pathway (Ugm1, Gt4c) (36); (ii) cell wall remodeling enzymes, including Gel and Bgt glucosyltransferases involved in the elongation and branching of the β1,3 glucan (37); and (iii) some enzymes involved in mannosylation, including multienzyme complexes mannan polymerase I and II involved in the synthesis of one of the two conidial mannans (Mnn proteins, Van1, Och1) (38) and Pmt O-mannosyltransferases involved in the 0-mannosylation of cell wall remodeling enzymes (39). Other cell wall-related proteins, including Mp1, PhiA, and MidA; glycosylphosphatidylinositol (GPI)-anchored proteins, including Ecm33; mannosyltransferases (Alg2, Och1and MnnII); and the putative glycan biosynthesis protein Pig1, were also detected. Of note, the proteins identified under nonregenerating conditions likely represent underestimations, as a consequence of the lower production of EVs under those conditions.

**TABLE 1:** Cell wall-associated proteins found in A. fumigatus EVs produced by protoplasts^*a*^

Condition	UniProt annotation	UniProt no.	Accession no.	Standard name(s)
Cell wall synthesis only	Probable glucan endo-1,3-beta-glucosidase EglC	B0XXF8	AFUB_048180	Bgt2
	Probable beta-glucosidase E BglE	B0YD91	AFUB_094720	Exg21
	Filament-forming protein (Tpr/p270), putative	B0XM26	AFUB_001640	
	Cell wall biogenesis protein Mhp1, putative	B0XR76	AFUB_012380	
	O-Methyltransferase	B0XVZ1	AFUB_033500	
	1,4-Alpha-glucan branching enzyme	B0Y0Q4	AFUB_058160	
	Alpha-1,2-mannosyltransferase (Alg2), putative	B0Y1U9	AFUB_060920	Alg2
	Alpha-1,6-mannosyltransferase subunit (Och1), putative	B0Y410	AFUB_056120	Och1
	Glycan biosynthesis protein (PigL), putative	B0YAG1	AFUB_084550	PigL
	Alpha-1,2-Mannosidase	B0Y765	AFUB_072720	
	Alpha-1,2-mannosyltransferase, putative	B0Y1T7	AFUB_060800	MnnII
	Alpha-N-acetylglucosamine transferase	B0YA98	AFUB_083900	

Cell wall repression only	Mannosylphosphorylation protein (Mnn4), putative	B0XN98	AFUB_004200	Mnn4
	GPI-anchored cell surface glycoprotein, putative	B0Y1D8	AFUB_059930	
	Chitin synthase activator (Chs3), putative	B0Y9Q8	AFUB_081930	Chs3
	Mannosyltransferase PMTI	B0YA13	AFUB_083000	Pmt4
	Probable glucan endo-1,3-beta-glucosidase BtgC	B0Y429	AFUB_056310	Bgt3
	Cell wall proline-rich protein, putative	B0XRJ9	AFUB_012940	
	Glycosyl hydrolase, putative	B0XYB1	AFUB_040280	

Both cell wall synthesis and cell wall repression	Chitin synthase, putative	B0XTD9	AFUB_029070	CsmB
	Chitin synthase ChsE	B0XTE0	AFUB_029080	ChsE
	Chitin synthase	B0XTK9	AFUB_018960	ChsA
	Chitin synthase activator (Chs3), putative	B0XZ75	AFUB_043410	Chs3
	Chitin synthase	B0XZY5	AFUB_034810	ChsG
	Chitin synthase F	B0Y9Q7	AFUB_081920	ChsF
	Class V chitinase, putative	B0YBH2	AFUB_092800	
	Chitin biosynthesis protein (Chs5), putative	B0YDJ8	AFUB_095840	Chs5
	Chitin biosynthesis protein (Chs7), putative	B0XQX5	AFUB_011500	Chs7
	Alpha-1,3-glucan synthase, putative	B0XNF7	AFUB_014990	Ags1/Ags2/Ags3
	Alpha-1,3-glucan synthase, putative	B0XX26	AFUB_047490	Ags1/Ags2/Ags3
	1,3-Beta-glucan synthase catalytic subunit FksP	B0Y8S7	AFUB_078400	Fksp/Fks1
	Cell wall protein PhiA	B0Y004	AFUB_045170	Aspf34
	Cell wall biogenesis protein phosphatase Ssd1, putative	B0XQR1	AFUB_010850	Ssd1
	Cell wall protein, putative	B0XXP9	AFUB_038170	MidA
	GPI-anchored cell wall protein, putative	B0Y688	AFUB_066060	
	GPI-anchored cell wall organization protein Ecm33	B0Y5M3	AFUB_063890	Ecm33
	GPI-anchored protein, putative	B0YDG5	AFUB_095500	
	Cell wall integrity signaling protein Lsp1, putative	B0Y7E0	AFUB_073480	Pil1
	Cell wall serine-threonine-rich galactomannoprotein Mp1	B0YEP2	AFUB_099880	Mp1
	1,3-Beta-glucanosyltransferase Gel1	B0XT72	AFUB_018250	Gel1
	1,3-Beta-glucanosyltransferase Gel4	B0XVI5	AFUB_022370	Gel4
	Mannan endo-1,6-alpha-mannosidase	B0XXF1	AFUB_048110	
	Alpha-1,6-mannosyltransferase subunit (Mnn9), putative	B0XTG8	AFUB_018530	Mnn9
	Dolichol-phosphate mannosyltransferase, putative	B0XXW0	AFUB_038750	
	Protein mannosyltransferase 1	B0XYZ3	AFUB_042600	Pmt1
	Alpha-1,2-mannosyltransferase (Kre2), putative	B0Y0S4	AFUB_058360	Ktr1
	Alpha-1,2-mannosyltransferase (Kre5), putative	B0Y1C4	AFUB_059750	Ktr7
	Alpha-1,2-mannosyltransferase (Ktr4), putative	B0Y2F5	AFUB_051270	Ktr4
	Alpha-1,6-mannosyltransferase subunit, putative	B0Y6R0	AFUB_067830	Mnn11
	UDP-glucose 4-epimerase	B0Y0S6	AFUB_058380	Uge5
	UDP-glucose:glycoprotein glucosyltransferase, putative	B0XTX7	AFUB_019450	
	Glycosyl transferase, putative	B0XYK7	AFUB_041250	Gt4b/Gt4c
	Glycosyl transferase, putative	B0YAG3	AFUB_084570	Och3
	Glycosyl transferase, putative	B0XZM8	AFUB_044600	
	N-Glycosyl-transferase	B0Y5M8	AFUB_063940	
	Lysophospholipase 3	B0XZV8	AFUB_034540	Plb3
	Lysophospholipase 1	B0Y665	AFUB_065820	Plb1
	Rho GTPase Rho1	B0Y776	AFUB_072830	Rho1
	1,3-Beta-glucanosyltransferase Bgt1	B0XQR5	AFUB_010890	Bgt1
	Cell wall glycosidase	B0XNL0	AFUB_015530	Aspf9/Crf1
	Cell wall glucanase, putative	B0XY72	AFUB_039870	Crh3
	Mannan polymerase II complex ANP1 subunit Anp1, putative	B0XUV6	AFUB_031580	Van1
	SUN domain protein (Uth1), putative	B0YCQ5	AFUB_091030	Sun1
	Probable beta-glucosidase BtgE	B0Y9Q9	AFUB_081940	BtgE/Sw11
	Putative UDP-galactopyranose mutase	B0XWU8	AFUB_036480	Ugm1
	Endo alpha-1,4 polygalactosaminidase, putative	B0XYK5	AFUB_041230	Ega3

a For threshold detection limits and false-discovery rates, please see Materials and Methods.

## DISCUSSION

Yeast forms of different fungal species produce extracellular membrane structures classified as EVs (40). More recently, it was demonstrated that filamentous forms of fungi also produce EVs (18–20, 22). Although the functional impact of these findings is still not clear, they confirm that EVs are released by fungi in different morphological stages as part of distinct physiological events. In most eukaryotes, EVs are released at the plasma membrane level. However, in fungi and plants, the cell wall is usually the outermost cell layer, increasing the complexity of understanding the physiological function of eukaryotic EVs. Our study has shown that living wall-less stages such as protoplasts are useful as models to analyze the role of fungal EVs in cell wall biogenesis. Of note, protoplasts of other species, including C. albicans, Schizosaccharomyces pombe, and Neurospora crassa, also produced extracellular particles resembling EVs (23, 27, 41), as concluded from previous microscopic analyses of fungal cells.

Our SEM analysis of protoplast forms of A. fumigatus germinating conidia revealed the presence of particles with general properties compatible with those of EVs being released from the plasma membrane, including morphology, dimensions, and bilayered membranes (42). These vesicular particles were morphologically similar to those observed in association with the cell wall of C. neoformans (43). This result and the observation of vesicles emerging from the A. nidulans surface (23) suggested that A. fumigatus protoplasts are efficient producers of EVs.

EVs released by regenerating protoplasts showed fibril-like material attached to the lipid surface, as revealed by TEM of isolated EVs. Even though the EVs were produced in the highest number in regenerating protoplasts, it is noteworthy that EV release was not uniquely associated with cell wall biosynthesis since the nonregenerating protoplasts also produced EVs. This observation suggests that EV release is not exclusively related to the synthesis of the cell wall and that the release of EVs in nongrowing cells may be also a response of the fungus to extracellular stress such high osmotic pressure or lack of nutrients. It is noteworthy that EVs from cell wall-regenerating cells and nonregenerating protoplasts differed in relative concentrations. Considering that these experimental systems correspond to conditions of nutrient abundancy and starvation, respectively, the quantitative differences could simply correspond to a more efficient metabolic response of nonstarved cells. Indeed, the impact of the nutritional availability on the production of microbial EVs has been reported before. In Mycobacterium tuberculosis, EV production was found to increase in response to iron restriction (44). Alternatively, the increased number of EVs in our protoplast model might indicate an association between vesicle production and cell wall synthesis. That supposition remains to be experimentally proved, but the finding that S. cerevisiae strains with deletions in cell wall biosynthesis genes produced more EVs than parental cells (45) argues against this hypothesis but favors the hypothesis that stress increases production of EVs.

Our carbohydrate analysis revealed the presence of Man and Gal, an increased amount of Glc, and the presence of GalNAc in EVs obtained from regenerating protoplasts. Glc is a marker of β1,3 or α1,3 glucan. Man and GaI are markers of galactomannan. Our current results suggest that the EV population produced by A. fumigatus includes plasma membrane-derived vesicles, as consistently described for the mammalian EVs denominated microvesicles (24). Therefore, we speculate that β1,3 or α1,3 glucan is incorporated in the EVs during their formation, as concluded from previous demonstrations that cell wall polysaccharides are synthesized on the internal side of the plasma membrane level and extruded in the cell wall at the C-terminal pore-like part of the respective enzymes (46, 47). Galactomannan is assembled in the Golgi apparatus and secreted to the plasma membrane before being cross-linked to β1,3 glucan, supposedly by extracellular transglycosidases (35). Therefore, the possible presence of glucans and galactomannan in EVs may be a consequence of their original association with the plasma membrane. GAG, which is a virulence-associated component of the A. fumigatus extracellular matrix (48, 49), is localized on the surface of the cell wall, where it acts as a component of the fungal extracellular matrix (50). The detection of GalNAc residues only in EVs obtained from regenerating protoplasts suggests that GAG is transported by vesicles through the cell wall to be deposited on the cell surface. Alternatively, GAG could be loosely associated with EVs, considering its “sticky” nature due to its great ability to form unspecific hydrogen bonds. Similar observations were described in C. neoformans, in which EVs were found to contain the extracellular polysaccharide glucuronoxylomannan (9).

It is still unknown whether the EVs characterized in our protoplast model represent the vesicular structures produced by intact A. fumigatus. In our study, we identified a higher diversity of EV proteins than of A. fumigatus mycelial vesicles (22), but the hypotheses explaining these differences are numerous. First, it is important to highlight that the strains used in these studies were distinct, which impairs an accurate comparison. Second, the proteomic analyses in these independent studies were performed, as usual in the literature, under very different technical conditions. Finally, all studies on fungal EVs produced so far had used distinct conditions for fungal growth that do not correlate with our model of nutritional abundancy (cell wall synthesis) or starvation (nonregenerating conditions). Compositional comparisons between this study and others are, therefore, of very limited applicability. Nevertheless, 32 of the 60 proteins described for the mycelial A. fumigatus EVs were also found in our study.

Our current results reinforce the idea that biogenesis of fungal EVs includes vesicle formation at the plasma membrane level, as demonstrated for other eukaryotes (24). However, the presence of intracellular sites of vesicle biogenesis and their relationship with the synthesis of the cell wall cannot be ruled out. For instance, Neurospora crassa chitin synthases 1, 3, and 6 were previously shown to be distributed into cytoplasmic vesicular compartments likely corresponding to chitosomes (51). In Zymoseptoria tritici, chitin and β(1,3)-glucan synthases were previously found to be coexported to the cell surface within the same vesicle (52). In plant cells, the glucan synthase-like protein NaGSL1 was detected in both intracellular vesicles and the plasma membrane, the latter location being associated with cell wall synthesis (53). In the *Aspergillus* model, protoplasts are produced from the germ tube tips, so they contained all the cell wall synthesis and remodeling machinery normally present in the plasma membranes of fungal apexes (36, 54, 55). Consequently, it is not surprising that protoplast EVs contained many of these cell wall enzymes. Some of these proteins, including Fks1, CsmB and Chs, Gel4, Pmt4, Ktr4, Ktr7, and Gt4C, are essential for the synthesis of the major cell wall polysaccharides β1,3 and α1,3 glucans, chitin, galactomannan, and GAG and for branching/elongation of the β1,3 glucan in A. fumigatus (56–58). The absence of GlcNAc (*N*-acetylglucosamine residues) may suggest that chitin-related molecules are absent in the EVs. However, we cannot rule out the possibility that a slower kinetics of chitin synthesis affected our experimental model. For instance, preliminary experiments of polysaccharide immunolabeling of regenerating protoplasts showed that β1,3 glucan was the first polysaccharide detected on the surface of the protoplasts, following by α1,3 glucan. Chitin was the last polysaccharide to be detected (A. Beauvais, unpublished results).

Proteins that are not predicted to be in the extracellular milieu were abundantly detected in EVs from A. fumigatus protoplasts. This observation agrees with numerous reports on the protein composition of fungal EVs (8, 11, 14, 31) and with the fact that the biogenesis of these membranous structures has been linked to the cytoplasm and the plasma membrane (59). Additionally, it was previously suggested that the cell wall is a storage site for many fungal proteins, including glucanases, PhiA, Ecm33, Gel1, and Gel4 (60–63).

Our current results contribute to a better understanding of the properties of fungal EVs. To our knowledge, this is the first characterization of EVs in protoplasts obtained from germinating conidia. Our results suggest that these cellular forms represent a promising model to explore novel roles of fungal EVs in many fungal species. In the A. fumigatus model, phagocytic cells stimulated with EVs increased their ability to produce inflammatory mediators and to promote fungal clearance (22). Similarly, A. flavus EVs affected the interaction between the fungus and host immune cells (64). These results support the relevance of the use of protoplastic fungal EVs to promote better understanding of their role in both the physiology and immunopathogenesis of A. fumigatus.

## MATERIALS AND METHODS

### Growth conditions and preparation of A. fumigatus protoplasts.

The A. fumigatus reference strain used in this study was CEA17ΔakuB^KU80^ (ku80), which is deficient in nonhomologous end joining (65). The CEA17ΔakuB^KU80^ strain was conserved on 2% (wt/vol) malt agar slants. Five-day-old conidia were recovered from the slants by vortex mixing performed with a 0.05% (vol/vol) aqueous Tween 20 solution and filtered through a 40-μm-pore-size cell strainer. For protoplast preparation, 7 × 10^9^ conidia suspended in 0.05% Tween were centrifuged at 3,000 × *g* for 10 min. The supernatant was discarded, and the cells were suspended in 10 ml of sterile water. The conidia were inoculated in 600 ml of germination medium (1% yeast extract, 3% glucose, and 0.6 M mannitol) and then incubated with shaking for 14 h at 30°C. Germinated conidia were harvested and separated from dormant conidia by filtering the sample through a sterile Miracloth-lined funnel and then washed with 200 ml of sterile osmotic medium (OM; 1.2 M MgSO_4_ · 7H_2_O, 0.09 M K_2_HPO_4_, and 0.01 M KH_2_PO_4_, pH 5.8) and subjected to 2-fold dilution. After the washing step, the germinated conidia were suspended in 20 ml OM containing Glucanex (Novo Nordisk Ferment Ltd., catalog number CH4243) at 30 mg/ml. The cells were gently homogenized, and the suspension was adjusted to a final volume of 250 ml with a filtered-sterile Glucanex solution for hydrolysis of the cell wall.

Cell wall digestion was performed in 2-liter Erlenmeyer flasks for 2 h at 37°C, with gentle shaking (60 rpm), until sufficient protoplasts were released, as assessed microscopically. For protoplast recovery, the cells were harvested by filtration in a sterile glass Buchner funnel (porosity 2). After filtration, 2 volumes of sterile 0.3 M KCl were added to 1 volume of the protoplast suspension in Glucanex. The mixture was centrifuged at 5,000 × *g* (20 min, 25°C). The pellet was suspended in sterile 0.6 M KCl and washed twice (3,000 × *g*, 10 min per wash, 25°C, with minimal break) to eliminate the remaining Glucanex. The final pellet of fresh protoplasts was divided into two equal parts. Each part was suspended in 10 ml of sterile 0.6 M KCl and further incubated under nonregenerating or cell wall synthesis conditions. The viability of the protoplasts was assessed by the use of trypan blue dye exclusion at different time points, from 0 to 2 h.

For cell wall regeneration, the protoplasts were incubated in 400 ml of a minimal medium (MM) supplemented with 0.6 M KCl for 2 h at 37°C, with shaking (120 rpm). The MM was prepared as previously described (66, 67) with some modifications and contained 1% (wt/vol) glucose, 20 mM glutamine, 0.052% KCl, 0.052% MgSO_4_ · 7H_2_O, 0.152% KH_2_PO_4_, and 1 ml trace element solution (pH 6.5). Alternatively, protoplasts were incubated for 2 h in a nonregenerating solution of 0.6 M KCl or were immediately processed for the analyses described below.

### Microscopic analysis of protoplasts.

Freshly purified protoplasts, as well as protoplasts obtained under cell wall-regenerating or nonregenerating conditions, were fixed with 2% formaldehyde–0.6 M KCl and stored at 4°C. For fluorescence or superresolution microscopy, the cells were washed twice with phosphate-buffered saline (PBS) and blocked with superblock blocking buffer (Thermo Scientific, catalog number 37515) mixed in PBS for 1 h at 37°C. Surface α-(1,3)-glucan of protoplasts was labeled with MOPC 104E monoclonal antibody (Sigma-Aldrich, catalog number M5909) (2 μg/ml; 1 h at 37°C) (68). Alternatively, the cells were stained with an anti-GAG monoclonal antibody (20 μg/ml; 1 h at 37°C) produced in the Latgé laboratory as previously reported (48). After two washes in PBS, each preparation was incubated with the appropriate secondary antibodies (anti-mouse IgM Alexa Fluor 488 for glucan staining; anti-mouse IgG Alexa Fluor 488 for GAG; both diluted 1:100 in blocking buffer). After incubation for 1 h at 37°C, the cells were washed three times with PBS. The protoplast membranes were finally stained with Vybrant DiI cell-labeling solution (Molecular Probes, catalog number V22885) at 5 μM (30 min, 37°C) and washed one final time with PBS. The cells were placed on glass slides covered with ProLong Gold antifade reagent. The cells were microscopically observed under a Zeiss Axioplan 2 fluorescence microscope or a Zeiss Elyra PS.1 superresolution microscope using structural illumination mode. Images were obtained with ZEN 2.1 software.

Superresolution scanning electron microscopy (SEM) was performed as previously described (69). Briefly, 10^6^ protoplasts were fixed on sterile glass coverslips (previously coated with poly-l-lysine) overnight at 4°C on 24-well plates. The samples were dehydrated in a graded ethanol series (30%, 50%, and 70% for 5 min and 95% and 100% for 10 min), subjected to critical point drying in CO_2_, mounted on stubs, and coated with carbon. Observation of the protoplast cell surface was performed with an Auriga 40 field emission scanning electron microscope (FE-SEM) microscope (Zeiss, Germany).

Protoplasts were also were analyzed with a JEOL JSM-6700F apparatus, which is an ultra-high-resolution field emission scanning electron microscope equipped with a cold-field-emission gun and a strongly excited conical lens. The secondary-electron image resolution settings were 1 nm at 15 kV and 2.2 nm at 1 kV. Pieces of culture were frozen using a Gatan Alto 2500 cryo-stage and cryo-preparation chamber. The preparation conditions were as described previously by Paris et al. (70).

### EV isolation and physical-chemical analysis.

Isolation of EVs from protoplast supernatants was performed as previously described for yeast cells (40), with minor modifications. Briefly, after each incubation period, the supernatants were separated from the protoplast cells by centrifugation at 3,000 × *g* (15 min, 25°C, with no brake) and sequentially passed through filters with 5-μm, 1.2-μm, and 0.45-μm pore sizes. The pellets containing protoplast cells were stored at −20°C for sterol quantification. After filtration, the supernatants were concentrated in an Amicon ultrafiltration system (cutoff, 100 kDa) and again centrifuged at 10,000 × *g* and 4°C for 15 min to eliminate possible cellular debris. The concentrated supernatants were finally ultracentrifuged at 100,000 × *g* (4°C, 1 h). The resulting pellets containing EVs were washed twice with filtered PBS (0.22-μm pore size) under the same ultracentrifugation conditions and finally suspended in 300 μl of filtered PBS (0.22-μm pore size). The EV suspensions were stored at −80°C for further experiments.

For nanoparticle tracking analysis (NTA) and GAG serological detection, the EV suspensions were first submitted to immunoprecipitation for the removal of nonvesicular polysaccharides. In this assay, 50 μl of the EV suspension was added to the wells of a 96-well enzyme-linked immunosorbent assay (ELISA) plate, previously coated with a mixture of antibodies against α-glucan (J558) (71), β-glucan [Dectin 1 human IgG Fc chimeric β‐(1,3)‐glucan receptor; a kind gift of G. Brown, University of Aberdeen, United Kingdom], and GAG (1 μg/ml, 1 h, 37°C) (48) and blocked with PBS containing 1% bovine serum albumin (BSA). Unbound fractions were collected, and the resulting EVs were stored at −80°C for further experiments.

Nanoparticle tracking analysis (NTA) was performed to determine the EV diameter and concentration. NTA of protoplast EVs was performed on an LM10 nanoparticle analysis system coupled with a 488-nm-wavelength laser and equipped with a scientific complementary metal oxide semiconductor (sCMOS) camera and a syringe pump (Malvern Panalytical, Malvern, United Kingdom), as recently described for C. gattii EVs (72). The samples were subjected to 25-fold dilution in filtered PBS and measured within the optimal dilution range of 7.6 × 10^7^ to 6.8 × 10^8^ particles/ml. The data were acquired and analyzed using NTA 3.0 software (Malvern Panalytical). NTA values were used to calculate ratios of EVs to cells by dividing the number of particles detected in the equipment by the number of cells in the original individual vesicle samples. For these analyses, two independent biological replicates were prepared and each sample was submitted to at least three reads, generating six measurements for each sample. These values were adjusted according to the original sample dilution (25-fold). The quantification of sterol in EV preparations was performed with an Amplex red cholesterol assay kit (14, 73, 74).

### Transmission electron microscopy (TEM) of EVs.

For negative-staining TEM, the EV pellets were fixed with 2% glutaraldehyde–2% paraformaldehyde–0.1 M sodium cacodylate buffer at room temperature for 2 h and then postfixed overnight at 4°C with 1% glutaraldehyde–4% paraformaldehyde–PBS. Copper carbon-coated grids (Cu-CF300; EMS), previously negatively charged by the use of an Elmo system (1 min, 15 pascals, 2 mA, 80 V), were put in contact with 15 μl of each sample for 10 min and washed three times with Milli-Q water drops (2 min each time), stained with uranyl acetate 2%, dried, and observed with a Tecnai Spirit microscope operating at 120 kV and equipped with an Eagle 4,000-pixel-by-4,000-pixel camera.

EVs were alternatively fixed with 2% formaldehyde–2% glutaraldehyde–cacodylate buffer (0.1 M, pH 7.4). The samples were washed through four changes of cacodylate buffer (30 min each) and pelleted in 1% agarose (JT Baker Chemical Co., Phillipsburg, NJ). They were transferred to 1% osmium tetroxide in cacodylate buffer (0.1 M, pH 7.4) and incubated at 4°C for 1 h followed by washing in cacodylate buffer and distilled water performed for a total of 30 min. The samples were then stained with 0.5% aqueous uranyl acetate, dehydrated, slowly infiltrated with epoxy, and embedded. After resin polymerization, the blocks were sectioned on a Leica ultramicrotome and subjected to poststaining for 10 min in 2% uranyl acetate–50% ethanol and for 5 min in lead citrate. Ultrathin sections (70 nm thick) were collected on Formvar-coated copper slot grids and subjected to poststaining for 10 min in 2% uranyl acetate–50% ethanol and for 5 min in lead citrate. Sections were then examined on a JEOL 1200EX microscope (JEOL Ltd., Tokyo, Japan) equipped with a SIA L3C charge-coupled-device (CCD) camera (SIA Inc., Duluth, GA).

### Monosaccharide composition and serological detection of GAG in EVs.

EV ultracentrifugation pellets were suspended in water for monosaccharide analysis. The presence of monosaccharides in the EVs was determined by gas chromatography after hydrolysis, reduction, and paracetylation of the vesicle components using meso-inositol as an internal standard (75). Serological estimation of vesicular GAG levels was performed as described before by our group for other fungal polysaccharides (74). Briefly, EV suspensions were vacuum dried and suspended in chloroform/methanol (9:1 [vol/vol]). The suspension was centrifuged, and the resulting white precipitate was solubilized in PBS for quantitative ELISA performed with the anti-GAG antibody. The purified polysaccharide (SGG; a kind gift of T. Fontaine, Institut Pasteur, Paris, France) was used for the preparation of standard curves and determination of polysaccharide concentrations in EV samples (48).

### Protein composition of EVs.

EV ultracentrifugation pellets were suspended in buffer containing 8 M urea–100 mM Tris (pH 7.5) for proteomic analysis. Protein samples were reduced with 5 mM dithiothreitol (DTT) for 30 min at 23°C and then alkylated with 20 mM iodoacetamide in the dark at room temperature, for 30 min. Subsequently, the endoproteinase LysC (Promega) was added for the first digestion step (protein-to-Lys-C ratio = 80:1) for 3 h at 30°C. The sample was then diluted to reach a 1 M urea concentration with 100 mM Tris (pH 7.5), and trypsin (Promega) was added to the sample (protein-to-trypsin ratio = 50:1). The samples were digested for 16 h at 37°C. Proteolysis was stopped by the addition of 1% formic acid (FA). The resulting peptides were desalted using a Sep-Pak SPE cartridge (Waters) according to the manufacturer’s instructions.

Analysis of digested peptides by the use of liquid chromatography coupled to tandem mass spectrometry (LC-MS/MS) was performed on an Orbitrap Q Exactive Plus mass spectrometer (Thermo Fisher Scientific, Bremen, Germany) coupled to an EASY-nLC 1000 liquid chromatograph (Thermo Fisher Scientific). The peptides were loaded and separated at 250 nl · min^−1^ on a homemade C_18_ 50-cm capillary column with a picotip silica emitter (Dr. Maisch GmbH, Ammerbuch-Entringen, Germany) (75-μm diameter filled with 1.9-μm-pore-size Reprosil-Pur Basic C_18_-HD resin) equilibrated in solvent A (0.1% formic acid). The peptides were eluted using a gradient of solvent B (acetonitrile [ACN], 0.1% FA) of from 2% to 18% for 110 min, 18% to 30% for 35 min, and 30% to 45% for 15 min under conditions of a 250 nl/min flow rate. The total duration of the chromatographic run was 185 min, including high-ACN-level steps and column regeneration. Mass spectra were obtained in data-dependent acquisition mode with Xcalibur 2.2 software (Thermo Fisher Scientific, Bremen) with automatic switching between MS and MS/MS scans using a top-10 method. Spectral resolution corresponded to 70,000 (at *m*/*z* 400) with a target value of 3 × 10^6^ ions. The scan range was limited from 300 to 1,700 *m*/*z*. Peptide fragmentation was performed via higher-energy collision dissociation (HCD), with the energy set at a normalized collision energy (NCE) value of 28. The intensity threshold for the ion selection was set at 1 × 10^6^ ions, with charge exclusion settings of *z* = 1 and >7. The MS/MS spectra were acquired at a resolution of 17,500 (at *m*/*z* 400). The isolation window was set at 1.6 Th. Dynamic exclusion was employed within 45 s.

A data search was performed with MaxQuant tool (76) (version 1.5.3.8) with the Andromeda search engine against the A. fumigatus A1163 database (9,942 entries, downloaded from https://www.uniprot.org [accessed 18 September 2019]). As search parameters, carbamidomethylation of cysteines was set as a fixed modification and oxidation of methionine and protein N-terminal acetylation were set as variable modifications. The mass tolerances in MS and MS/MS were set to 5 ppm and 20 ppm, respectively. The maximum peptide charge value was set to 7, and 7 amino acids were required as the minimum peptide length. A false-discovery rate of 1% was set for both protein and peptide levels. Four independent EV ultracentrifugation pellets were analyzed for protoplasts submitted either to cell wall-regenerating conditions or repressing conditions. Two EV samples of freshly purified protoplasts were analyzed. Proteins identified in at least two independent experiments were assigned for Gene Ontology (GO). GO term enrichment was performed using the GOtermfinder feature of the AspGD database (http://www.aspergillusgenome.org/cgi-bin/GO/goTermFinder). Enriched GO terms were summarized by removing redundancy using the REVIGO Web server, available at http://revigo.irb.hr/ (77). Protein identification was based on the detection of one representative peptide by mass spectrometry after protein digestion. For sample preparation and qualitative comparison, EV samples were obtained from cultures with similar inocula and volumes. Revigo outputs were viewed in R (v4.0.0) using the treemap package. Treemap construction was based exclusively on proteins shared by all replicates.

### Statistical analysis.

All statistical analyses were performed using GraphPad Prism 6 software (GraphPad Software Inc.). Data sets were tested for normal distribution using Shapiro-Wilk or Kolmogorov-Smirnov normality tests. In the cases in which the data passed the normality test (alpha = 0.05), they were further analyzed using the unpaired Student's *t* test. Multiple data sets were further analyzed using ordinary one-way analysis of variance (ANOVA), followed by the Tukey’s multiple-comparison test. When at least one data set was nonnormally distributed, multiple data sets were analyzed by the nonparametric Kruskal-Wallis test.

### Data availability.

Accession numbers for proteins described in this work are available in Table 1. We confirm that the data supporting the findings of this study are available within the article and its supplemental material. Additional data supporting our findings are available from J.R. or M.L.R. upon reasonable request.
