# Supplementary figures and images for: Characterization of Extracellular Vesicles Produced by Aspergillus fumigatus Protoplasts
Source: mSphere. 2020 Aug 12;5(4):e00476-20. doi: 10.1128/mSphere.00476-20 (PMC7426166; doi:10.1128/mSphere.00476-20)

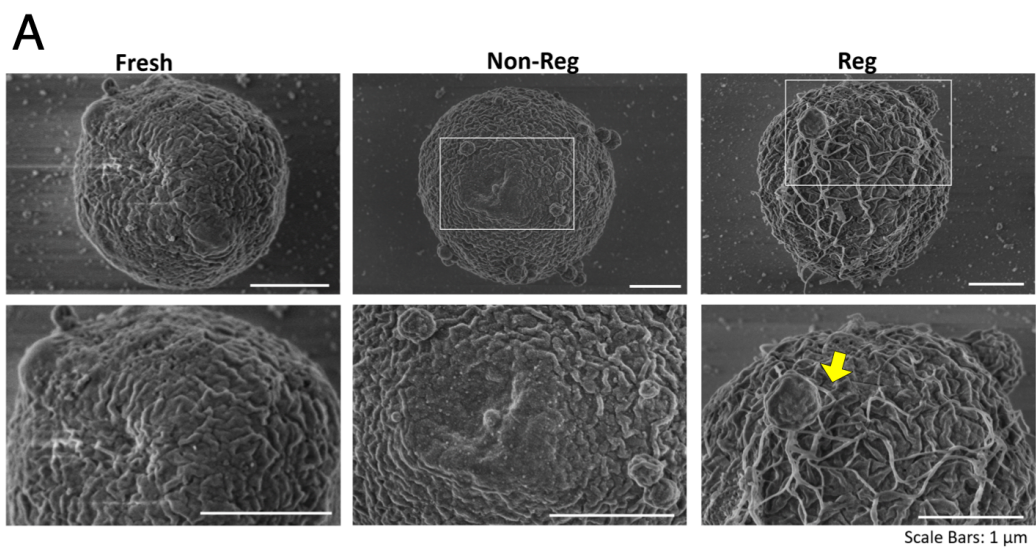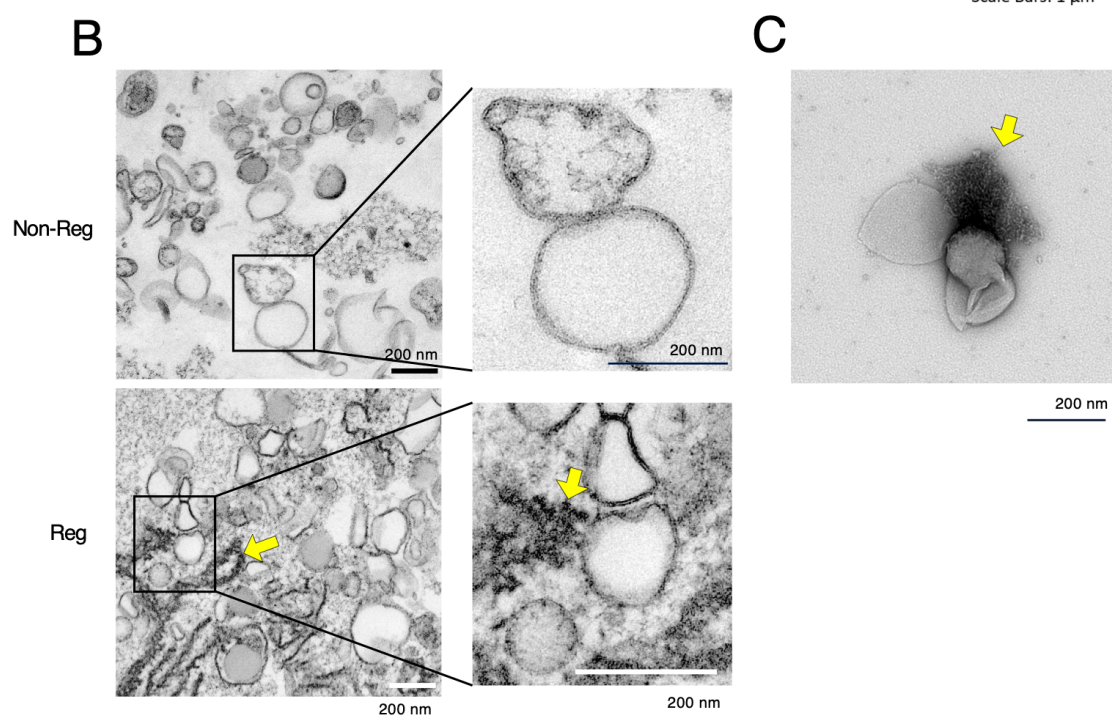

Supplement: FIG S1 [file mSphere.00476-20-sf001.pdf]
